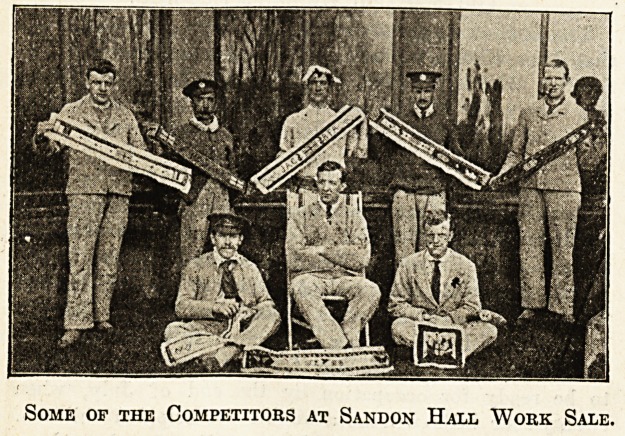# Auxiliary and Other Military Hospitals

**Published:** 1916-06-10

**Authors:** 


### Auxiliary and other Military Hospitals.

SANDON HALL, STAFFORD.

GREAT excitement prevailed in the Sandon Hall Military Hospital on May 19, the day fixed for the sale of work

SOME OF THE COMPETITORS AT SANDON HALL WORK SALE.

done by the patients. A glorious day broke—so hot that stalls and tea-tables were placed under the trees, making the grounds look like a foreign tea-garden. There were four stalls, one completely piled with the men's own handiwork in the shape of belts, baskets, rugs, fancy-work, etc., all finding ready purchasers. Another stall was for work by the staff, and a third of gifts sent by patients' friends. A fourth stall was covered with beautiful belts, all competing for a special prize. The work was judged by the Countess of Dartmouth, President of the Staffordshire Red Cross, assisted by Mrs. Bloomer, and their task was no easy one. A unique feature amongst the work was a fourfold screen, in cross-stitch, made by four of the patients, which won the first prize and realised nearly £6 in raffle tickets; it was won by the Countess of Powerscourt. The illustration shows a few of the men with their work. Sandon Hall has 105 beds, and was opened in September last, since when it has been always full. The men much appreciate its beautiful grounds, where they play cricket, tennis, bowls, etc., and are all one happy family. The sale realised about £70, which has been put to the Entertainment Fund.

[*** We should welcome short accounts of the life and doings at similar hospitals throughout the country. What are voluntary hospitals and convalescent camps doing in the same direction?—ED. THE HOSPITAL.]

## Figures and Tables

**Figure f1:**